# From “Every Day” Hormonal to Oxidative Stress Biomarkers in Blood and Follicular Fluid, to Embryo Quality and Pregnancy Success?

**DOI:** 10.1155/2019/1092415

**Published:** 2019-11-26

**Authors:** Katarzyna Olszak-Wąsik, Anna Bednarska-Czerwińska, Anita Olejek, Andrzej Tukiendorf

**Affiliations:** ^1^Department of Gynecology, Obstetrics and Oncological Gynecology, Silesian Medical University, Batorego 15, 41-902 Bytom, Katowice, Poland; ^2^GynCentrum Clinic, Żelazna 1, 40-851 Katowice, Poland; ^3^Department of Public Health, Wrocław Medical University, Bartla 5, 51-618 Wrocław, Poland

## Abstract

**Background:**

Oxidative stress appears to be involved in oocyte growth and maturation that when impaired results in poor embryo quality and lower potential to implant. The biochemical microenvironment of the oocyte (follicular fluid (FF)) consists of hormones and other various substances regulating the balance between oxidants and antioxidants.

**Aim:**

The aim of this study was to examine the possible impact of selected biomarkers (“every day,” hormonal biomarkers, enzymatic and nonenzymatic antioxidants, and also oxidative stress markers) in serum and FF, on embryo quality and pregnancy success in infertile women undergoing infertility treatment.

**Methods:**

All 53 patients, mean age 34.7 ± 4.1 years, with serum AMH level ≥ 0.7 ng/mL, were diagnosed with idiopathic infertility. They were stimulated in short antagonist protocol, followed by in vitro fertilization (IVF-ICSI intracytoplasmatic sperm injection) and a single embryo transfer. Follicular fluid was aspirated from the first mature follicle. In statistical analyses the R software was used, then all data was assessed with the Shapiro-Wilk test, logistic regression, and later the receiver operating characteristic (ROC) curve was applied using “pROC” R package.

**Results:**

We did not observe any correlation between AMH and embryo quality and pregnancy rate. Statistically significant results were only found for biomarkers examined in follicular fluid. Greater levels of GPX in FF were associated with the increased chance of producing a high quality embryo (the optimal cut-off concentration was established at over 450 lU/L.) Regarding pregnancy success, increasing levels of GR (cut-off at 21 IU/L), CuZnSOD (cut-off at 9NU/mL), and GST (cut-off at 2.5 IU/L) resulted in lower chances of a successful pregnancy.

**Conclusion:**

Our results indicate that FF markers may have some advantages in predicting embryo quality and pregnancy over AMH. The GPX system seems to be mostly related to embryo quality and pregnancy.

## 1. Introduction

Approximately 8-12% of couples worldwide suffer from the failure to establish a clinical pregnancy after 12 months of regular and unprotected sexual intercourse [1]. Although about 70% of the cases of infertility may be explained by female infertility such as ovulation problems (polycystic ovary syndrome, thyroid problems, and premature ovarian failure) or male infertility (poor-quality semen, a lack of sperm, and ejaculation disorders) [2], the cause of up to 30% of infertility cases is unknown [3].

Among infertile women, one of the main causes of low IVF success rates is related to impaired oocyte growth and maturation resulting in poor embryo quality and decreased potential to implant [4]. Although in vivo and in vitro studies indicate that various physiological and technical factors [5–11] impact embryo quality and pregnancy success, in clinical practice, many steps for the diagnosis and treatment of infertility are based on unspecific biomarkers, leading to less desirable outcomes [12].

Oxidative stress appears to be involved in defective oocyte development [13] but, although reports have shown that increased reactive oxygen species (ROS), high lipid peroxidation (LPO), and decreased total antioxidant capacity (TAC) in follicular fluid (FF) correlate with poor embryo quality and fertilization rates [14], no consensus has yet been achieved in this matter. Fluctuations of hormones and other various substances in FF, which forms the biochemical microenvironment of the oocyte [15, 16], have direct effects on maturation and quality of oocytes and, therefore, may be used as potential markers for predicting IVF success [17].

The aim of this study was to examine the possible impact of selected biomarkers (“every day,” hormonal biomarkers such as AMH (anti-Müllerian hormone), FSH (follicle-stimulating hormone), LH (luteinizing hormone), estradiol, progesterone, enzymatic and nonenzymatic antioxidants, and also oxidative stress markers) in serum and FF, and on embryo quality and pregnancy success in infertile women undergoing infertility treatment.

## 2. Material and Methods

### 2.1. Individuals and Analyses

The study was designed as a prospective study including 53 infertile women who sought medical attention at GynCentrum, Katowice, Poland, in 2017. All included patients aged between 27.2–46.0 years (mean 34.7 ± 4.1) with BMI ranged between 16.9 and 28 (mean 22.2 ± 2.8) were diagnosed with idiopathic primary infertility [18] . The duration of infertility ranged between 2 and 5 years. Hormonal serum results performed on day 3 of the menstrual cycle were FSH 6.87 ± 1.74 (ranged 2.84–11.80) mIU/mL, LH 6.12 ± 2.16 (ranged 2.77–11.83) mIU/mL, and AMH 3.68 ± 2.58 (ranged 0.70–12.31) ng/mL.

We excluded cases with endometriosis (based on medical history, transvaginal ultrasonography results, and ESHRE 2013 Guidelines [19]), polycystic ovary syndrome (based on medical history and examination and hormonal results according to ESHRE 2019 Guidelines [20]), and any chronic diseases, e.g., autoimmunological that could additionally influence their fertility. Smokers and alcohol users were excluded. All patients with $\text{anti}‐\text{M}\ddot{\text{u}}\text{llerian hormone }\left(\text{AMH}\right)\geq 0.7$ ng/mL were scheduled to undergo controlled ovarian hyperstimulation using the antagonist protocol, intracytoplasmic sperm injection, and a single embryo transfer. The starting dose of gonadotrophins for COH (controlled ovarian hyperstimulation) was based on age, serum AMH, serum FSH (follicle-stimulating hormone), and BMI. Gonadotropin (Gonal F, Merck Serono, UK, and Menopur, Ferring, Germany) doses were further adjusted according to ultrasound findings and estradiol measurements during stimulation monitoring.

### 2.2. Follicular Fluid Collection and Biomarkers Analysis

Ovarian pick-up was performed 36 hours after r-hCG injection (recombinant human chorionic gonadotropin, Ovitrelle, Merck Serono, UK). Follicular fluid from the first mature follicle aspirated was collected before any flushing for oocyte recovery. The FF was then centrifuged at 3 000 cycles/min and stored at -60°C until assayed. All samples were defrosted only once.

All laboratory hormonal measurements in FF (AMH (anti Mullerian hormone), FSH (follicle-stimulating hormone), LH (luteinizing hormone), estradiol, and progesterone) were performed using the electrochemiluminescent (ECLIA) immunoanalyser (Cobas e411, Roche Diagnostics, Mannheim, Germany).

In FF, glutathione peroxidase (GPX), glutathione reductase (GR), and glutathione S-transferase (GST) activities were measured by methods presented by Kasperczyk et al. [21]. For GPX activity, the kinetic method by Paglia and Valentine was used [22]. The reaction between reduced glutathione and t-Butyl hyperoxide is catalysed by GPX. NADPH-dependent glutathione reductase (GR) converts oxidised glutathione back to reduced glutathione. The GPX activity (in micromoles of NADPH oxidised per minute per gram protein) is proportional to the decreased absorbance at 340 nm. GR was measured according to Richterich [23]. GST was measured according to Habig and Jakoby [24]. The method of Oyanagui was used to measure the activity of SOD and isoenzymes MnSOD and CuZnSOD. The enzymatic activity of SOD was expressed in nitric units. The activity of SOD is equal to 1 nitric unit (NU) when it inhibits nitric ion production by 50% [25]. The concentration of thiol groups was determined following methods by Koster et al. [26]. The measurements of protein level in FF were performed on the A25 biochemistry analyser (BioSystems S.A., Barcelona, Spain) according to the manufacturer's instructions.

Measurements of catalase activity, total oxidant status, and total antioxidant capacity were conducted in an automated PerkinElmer analyser. Catalase activity was measured by the method of Johansson and Borg [27]. The activity of Cat-Px was showed as U/g protein.

For total oxidant status, we used methods presented by Erel [28]. The results are shown in *μ*mol/L.

Total antioxidant capacity (TAC) presented in mmol/L was measured according to Erel [29]. OSI was counted as the ratio of TOS to TAC.

For ceruloplasmin determination, methods of Richterich were applied [23]. Absorbance was measured at a wavelength of 546 nm and the values shown were in mg/dL.

The level of malondialdehyde (MDA) in FF was measured according to Ohkawa et al. [30]. Concentrations were given in *μ*mol/L. The lipofuscin (LPS) concentration was measured according to Jain. Values were shown as relative units (relative fluorescence lipid extract (RF)) [31].

### 2.3. Assessment of Embryo Quality

Only mature oocytes (MII oocytes, i.e., metaphase II oocytes) were used for intracytoplasmic sperm injection (ICSI). Sperm analysis fulfilled the criteria for WHO 2010 reference values [32]. Every day, from day 0 (day of fertilization) till the day of embryo transfer (day 3), the embryos were cultured in Vitrolife G1 medium and everyday assessment of embryo quality was based on ESHRE recommendations (ESHRE Atlas of Human Embryology [33]).

All patients signed an informed consent form approved by the Silesian Medical University Ethics Committee (ref. no. KB1/63/16). All methods were performed in accordance with the guidelines of the European Society of Human Reproduction & Embryology, American Society for Reproductive Medicine, and The Polish Society of Reproductive Medicine & Embryology.

Clinical characteristics of patients are reported in Table 1 (levels are presented as mean ± standard deviation and minimum–maximum).

### 2.4. Statistical Analysis

**Figure 1:**
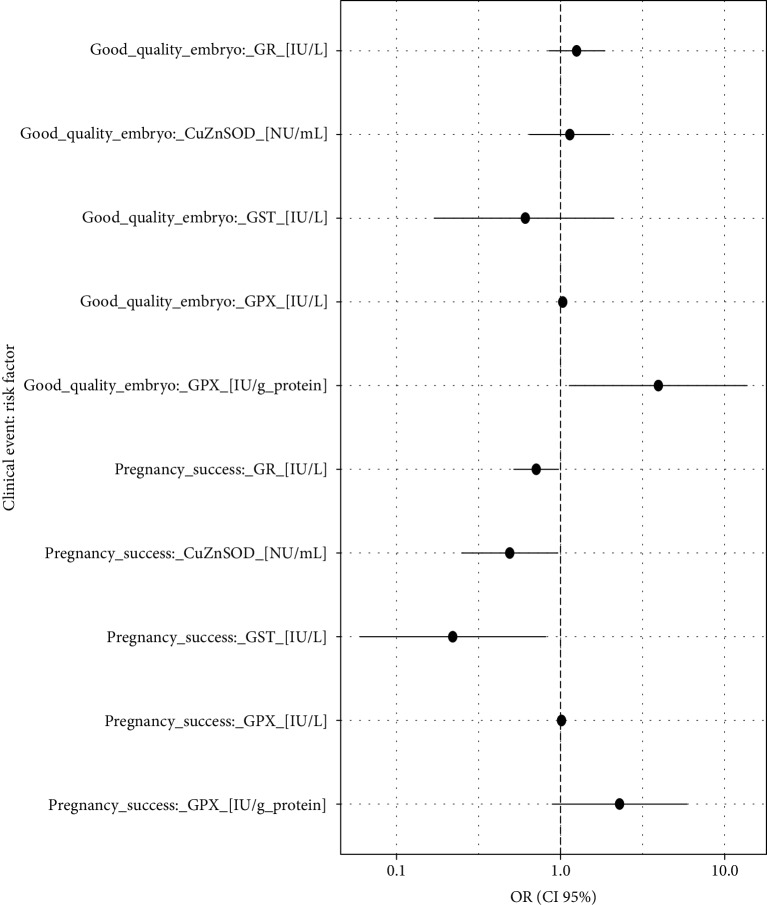
Forest plot of the ORs (pairs of biomarkers with statistically significant results). Statistical interpretation: an augmentation in the oxidative level in the FF, e.g., by 10 lU/L generates a (1.03^10^ − 1)∗100% = 34% increase in the chance of producing a high-quality embryo, i.e., by one-third.

Statistical analyses were performed using the R software [34]. All data were assessed for normality of distribution using the Shapiro-Wilk test. First, the difference of means between NTQ and TQ as well as pregnancy failure and success for the analysed biomarkers was estimated using classical Student's *t*-test for independent samples. To evaluate the possible impact of the selected biomarkers on the quality of embryos and pregnancy success, a logistic regression was applied, and the estimates were expressed by a classical odds ratio (OR). Only pairs of ORs with the statistically significant results (*P* < 0.05) for a good quality embryo and pregnancy success were reported and presented graphically in a forest plot (Figure 1). Additionally, the outcomes of interest were displayed graphically in the probability/frequency plots using “popbio” R package [35]. In these figures, a red line against a biomarker's concentration represents the curve of probability (left vertical axis) of analysed clinical events (good quality embryo and pregnancy success). Additionally, the graphs display the frequency of patients for each of the risk factors' intervals using grey columns, respectively, for negative and positive responses.

To determine the analysed ROS and hormone levels in FF that can discriminate between good quality and poor-quality embryos as well as pregnancy success and nonpregnancy events (best relation between sensitivity and specificity), the receiver operating characteristic (ROC) curve was applied using “pROC” R package [36]. The ROC curves were constructed by plotting the 1 − specificity (false positive rate) on the *x*-axis and the sensitivity (true positive rate) on the *y*-axis. The calculation of the area under the curve (AUC) was additionally made which measures the accuracy, i.e., the ability of the ROS and hormone value to discriminate between good and poor-quality embryos as well as pregnancy success and nonpregnancy events.

## 3. Results

The group of 53 analysed patients with idiopathic primary infertility was homogenous as showed in Table 1. The existing differences in top quality/nontop quality embryos and pregnancy success/failure were not numerous. The obtained results in Table 1 suggested analogous results in further ROC analysis of GR, CuZnSOD, GST, GPX, and GPX/g protein. Statistically significant results were only found for biomarkers examined in the follicular fluid. Greater levels of GPX, both at UI/L or UI/g protein, in FF were associated with the increased chance of producing a high-quality embryo (*P* = 0.03; Table 2). A 50% increase in the chances of producing high-quality embryos is achieved by an augmentation of 13.7 IU of GPX/L or 0.30 IU of GPX/g protein. Regarding pregnancy success, increasing levels of GR, CuZnSOD, and GST resulted in lower chances of a successful pregnancy (*P* ≤ 0.04; Table 2). An augmentation of GR, CuZnSOD, and GST by 2.1 IU/L, 0.98 NU/mL, and 0.47 IU/L, respectively, would result in a 50% lower chance of having a successful pregnancy. All other parameters evaluated for embryo quality and pregnancy success were not statistically significant (*P* > 0.05; Table 2).

**Figure 2:**
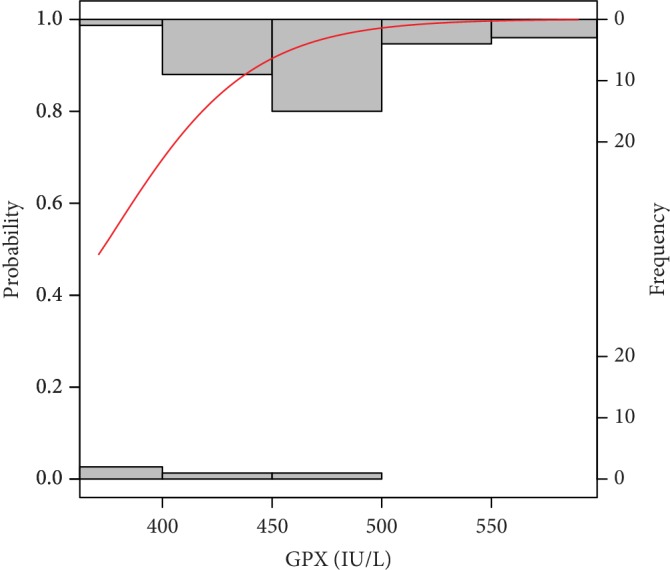
Probability model of good-quality embryo by GPX. Statistical interpretation: the modelled probability (*y*-axis) curve of a good-quality embryo (red line) increases rapidly with the concentration of GPX (*x*-axis) reaching a 95% chance of a good-quality embryo over 472 units.

**Figure 3:**
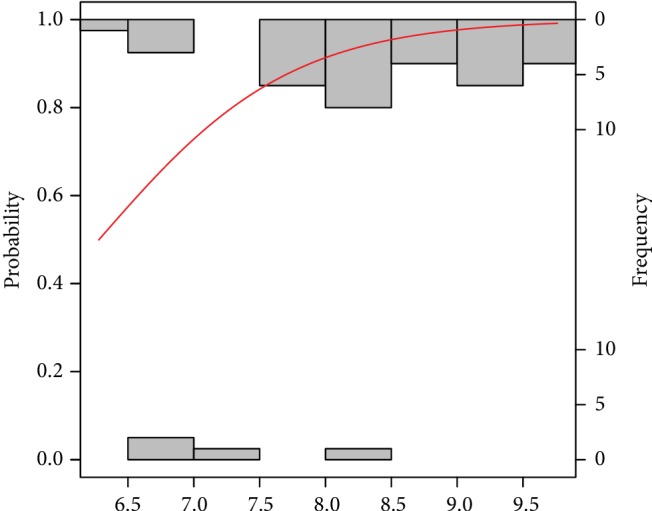
Probability model of a good-quality embryo by GPX in protein. Statistical interpretation: the modelled probability (*y*-axis) curve of a good-quality embryo (red line) increases rapidly with the concentration of GPX/g protein (*x*-axis) reaching a 95% chance of a good-quality embryo over 8.5 units of GPX/g protein.

**Figure 4:**
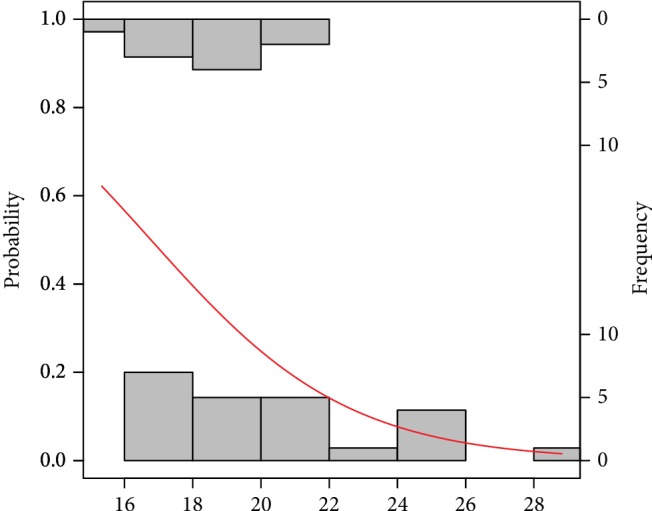
Probability model of pregnancy success by GR. Statistical interpretation: the modelled probability (*y*-axis) curve of the pregnancy (red line) decreases rapidly with the concentration of GR (*x*-axis) reaching no more than 50% chance of pregnancy over 17 units.

**Figure 5:**
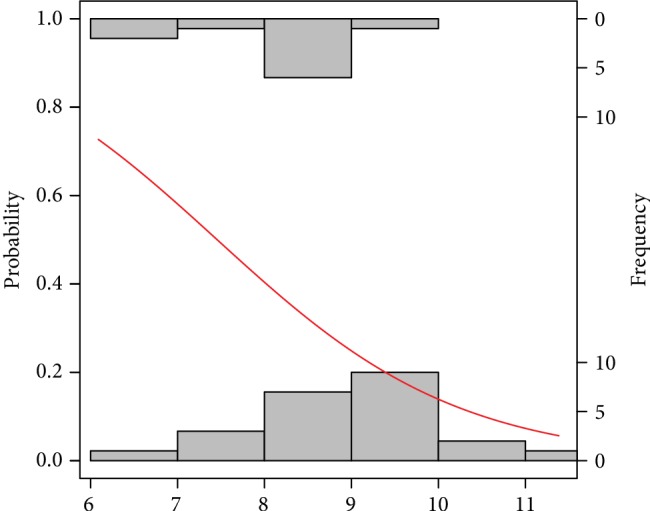
Probability model of pregnancy success by CuZnSOD. Statistical interpretation: the modelled probability (*y*-axis) curve of a pregnancy (red line) decreases rapidly with the concentration of CuZnSOD (*x*-axis) reaching no more than 50% chance of pregnancy over 7.5 units CuZnSOD.

**Figure 6:**
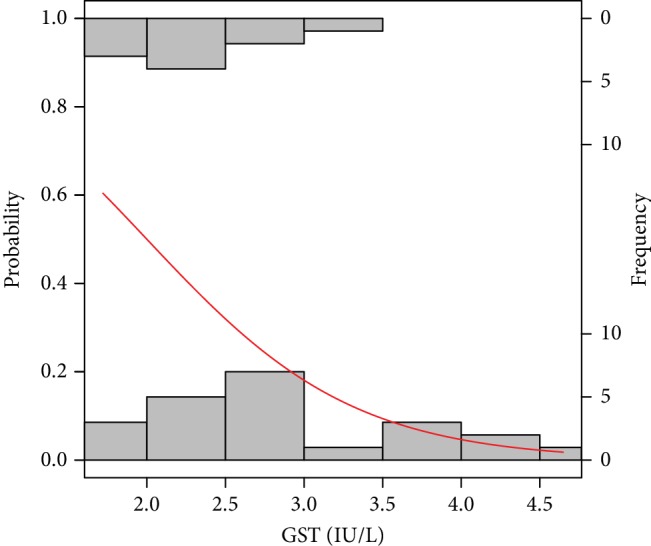
Probability model of pregnancy success by GST. Statistical interpretation: the modelled probability (*y*-axis) curve of a pregnancy (red line) decreases rapidly with the concentration of GST(*x*-axis) reaching no more than 50% chance of pregnancy yet over 2.0 units of GST.

**Figure 7:**
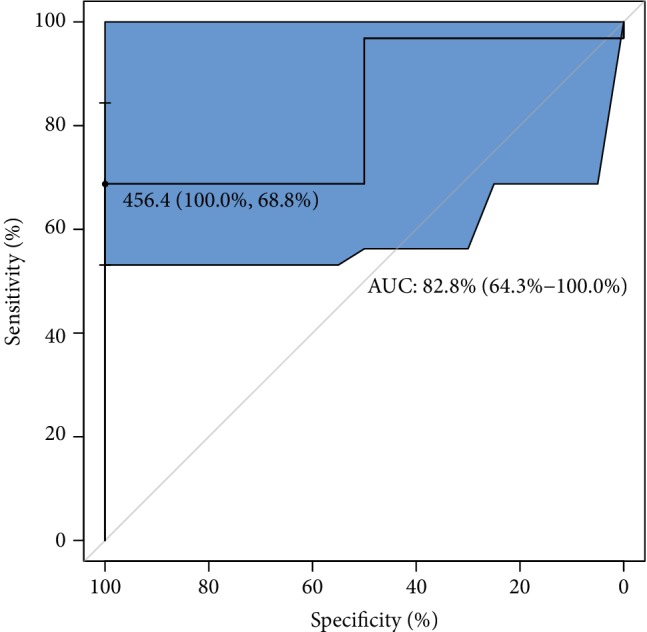
ROC analysis for classification of a good-quality of embryo by GPX. Statistical interpretation: the optimal cut-off of GPX concentration (maximum discrimination) to predict the good quality of an embryo was established over 450 lU/L. The estimated maximal specificity and sensitivity = 69% are relatively high, as well as AUC = 83%. This means that approximately 8 out of 10 good-quality embryos can be correctly predicted using the GPX concentration value.

The probability models obtaining good and poor-quality embryos as well as pregnancy success and a nonpregnancy event by the analysed risk factors are graphically presented in Figures 2–7. It can be seen in Figure 1 that the modelled probability (*y*-axis) curve of the good-quality embryo (red line) increases rapidly with the concentration of GPX (*x*-axis) reaching a 95% chance of a good-quality embryo over approximately 472 units. The shape of the modelled probability curve is derived from the OR given in Table 2. An almost identical course of the curve can be seen for the GPX/protein in Figure 3. In this case, the chance of more than a 95% occurrence of a good-quality embryo appears at a biomarker concentration greater than approximately 8.5 IU/g. In contrast, glutathione reductase is an effective negative predictor of pregnancy success. Its concentration > 17 IU/L provides evidence of a statistically significant minimal chance of fertilization (i.e., <50%) in the treated women (see Figure 3). The same patterns of probability can be seen for CuZnSOD and GPX in protein with a minimal chance of pregnancy starting from approximately 7.5 NU/mL and 2 IU/g of the biomarkers, respectively (Figures 4 and 5).

**Figure 8:**
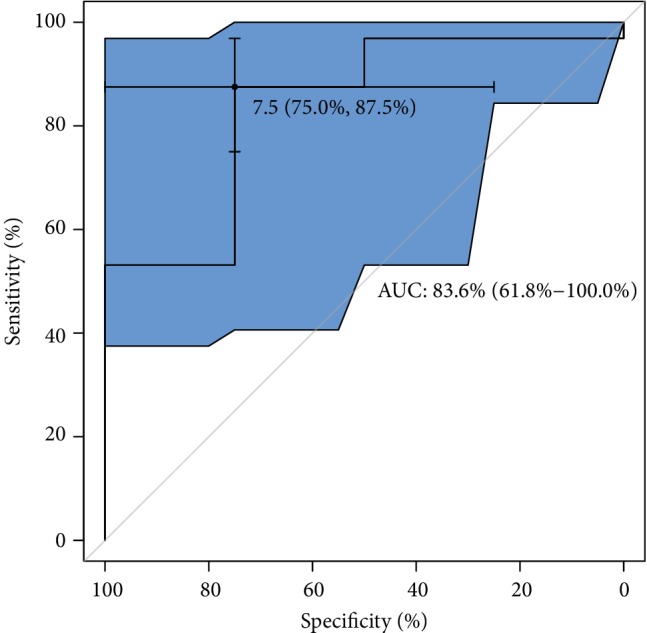
ROC analysis for classification of a good-quality of embryo by GPX in protein. Statistical interpretation: the optimal cut-off of GPX concentration (maximum discrimination) to predict the good quality of an embryo was established over 7.5 lU/g of protein. The estimated specificity = 75% and sensitivity = 88% are relatively high, as well as AUC = 84%. This means that approximately 8 out of 10 good-quality embryos can be correctly predicted using the GPX/g protein concentration value.

**Figure 9:**
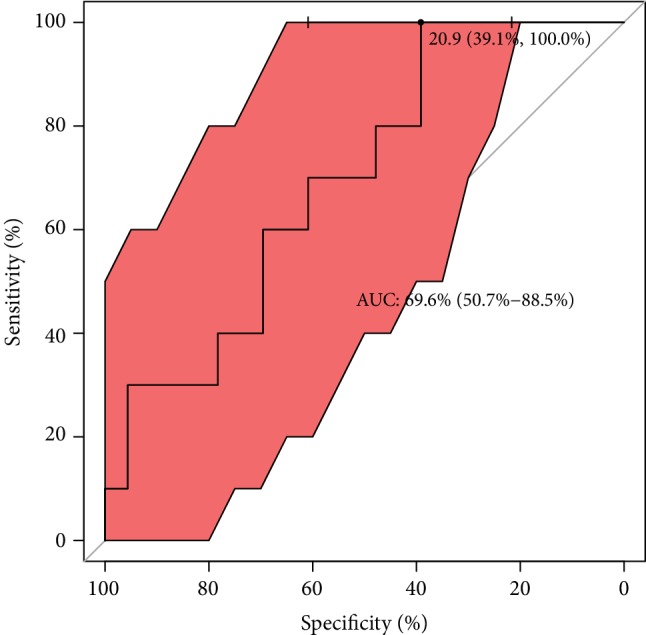
ROC analysis for classification of pregnancy success by GR. Statistical interpretation: the optimal cut-off of GR concentration (maximum discrimination) to predict a pregnancy was established to be under 21 lU/g. The estimated specificity = 39% is relatively low but sensitivity reached maximal prediction; AUC = 70% means that approximately 7 out of 10 pregnancy cases can be correctly predicted using the GR concentration value.

**Figure 10:**
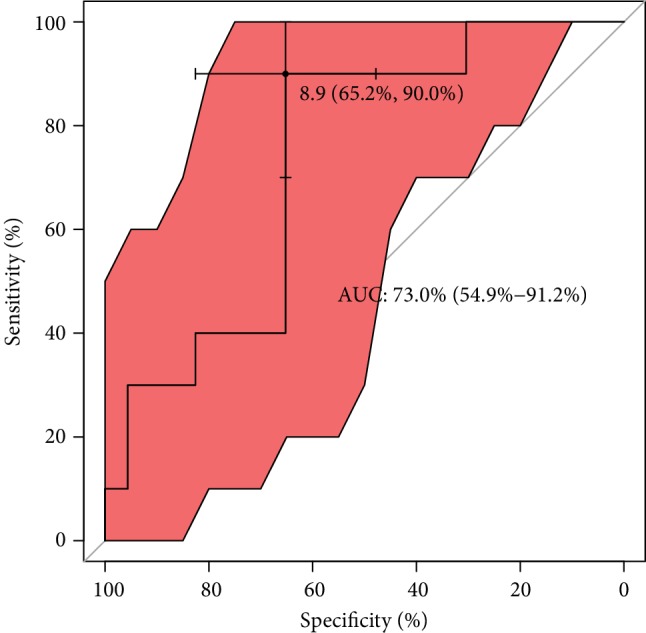
ROC analysis for classification of pregnancy success by CuZnSOD. Statistical interpretation: the optimal cut-off of CuZnSOD concentration (maximum discrimination) to predict if a pregnancy was established is under 9 NU/mL. The estimated specificity = 66% and sensitivity = 90% stands for a good prediction of the outcome; AUC = 73% means that nearly three-fourths of patients can be correctly predicted using the CuZnSOD concentration value.

**Figure 11:**
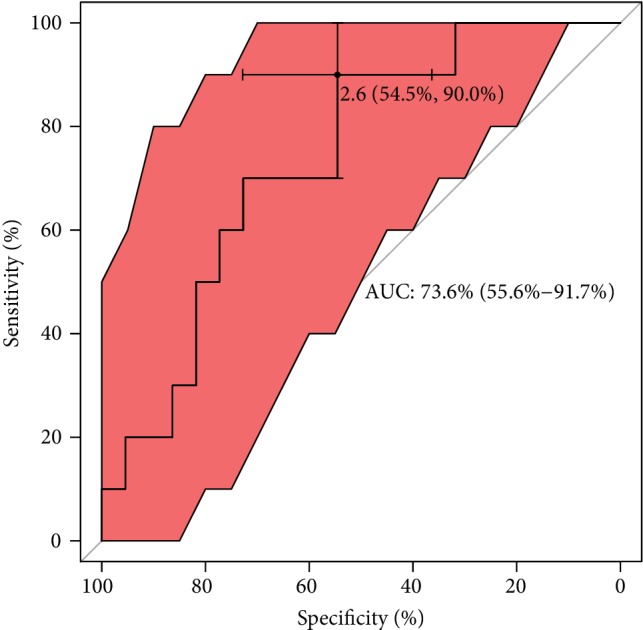
ROC analysis for classification of pregnancy success by GST. Statistical interpretation: the optimal cut-off of GST concentration (maximum discrimination) to predict if a pregnancy was established is under 2.6 lU/L. The estimated specificity = 55% is relatively low but sensitivity reached satisfactory prediction = 90%; AUC = 74% means that nearly three-fourths of patients can be correctly predicted using the GST concentration value.

With regard to ROC analysis, it can be seen in Figure 7 that the optimal cut-off of GPX concentration (maximum discrimination) to predict good quality of an embryo was established over 450 lU/L. Moreover, from clinical and diagnostic points of view, the estimated maximal sensitivity and specificity = 69% are relatively high, as well as AUC = 83%, and all this ROC analysis provides evidence of usefulness of the GPX testing in terms of the prediction of a good-quality of embryo in infertile women. Translating this into a practical language, this means that approximately 8 out of 10 good-quality embryos can be correctly predicted using the GPX concentration value. ROC estimates printed in Figure 8 show that GPX in protein has a similar level of embryo quality prediction (AUC = 84%), but with much higher sensitivity = 75% and specificity = 88%. Analogously, a poorer prediction of pregnancy success can be achieved using GR concentration (only 7 out of 10 fertilization outcomes can be correctly expected). The biomarker shows weak sensitivity (only 39%), however maximal specificity. The cut-off GR concentration was estimated at 21 IU/L (see the values printed in Figure 9). The remaining analysed two biomarkers CuZnSOD and GST give the very similar ROC outcomes (Figures 10 and 11). Using them, we cannot expect a prediction larger than three-fourth with much better specificity = 90% than sensitivity, at the cut-off points of approximately 9 NU/mL and 2.5 IU/L for CuZnSOD and GST, respectively. Finally, the correlation between a good-quality embryo and pregnancy success was calculated; however, the relationship was insignificant (*P* > 0.05).

## 4. Discussion

It is assessed that around 25% of patients experience unexplained infertility, where there is no apparent cause for a woman's infertility and the male factor is also excluded. A comprehensive review of the scientific literature reveals that the roles of different markers in various studies on the endpoints of embryo and pregnancy are controversial due to the differences in the nature of materials examined. Elimination of factors that could presumably lead to bias would provide support for an evidence-based approach [37].

### 4.1. AMH

AMH is widely used as a hormonal factor in determining ovarian reserve. In a nondirect way, it provides information about the quality of the oocyte. Patients with lower serum AMH tend to have worse results in the number and the quality of the retrieved eggs and then lower quality embryos [38]. Generally, embryo quality is determined by the number and regularity of blastomeres and the degree of embryonic fragmentation. Besides the morphological assessment of the embryo quality, visualisation of the dynamics in embryo development is used as an additional tool in the process of embryo selection [39].

In our study of infertile patients (idiopathic infertility), we found no AMH influence on embryo quality. In all our patients, the level of AMH was over 0.7 ng/mL. The rate of top quality embryos did not differ in groups with lower levels of AMH (rang 0.7-1.1 ng/mL) and higher (above 1.1 ng/mL). As stated by Grisendi et al., a cut-off value of AMH ranging between 0.7 and 1.3 ng/mL may be considered acceptable for the prediction of poor response in IVF [40]. Studies on the ability of AMH to predict oocytes' quality and live births are controversial and some suggest that AMH alone is a weak predictor of live birth after ART [40]. We did not observe a correlation between AMH and pregnancy rate. No prediction concerning embryo quality and pregnancy for other analysed hormonal factors were found.

### 4.2. Oxidative Stress Biomarkers

The average pregnancy rate per cycle in assisted reproduction is only 30%-40% [41]. Among the many reasons for the IVF failure, oxidative stress is an important factor [42]. ROS which are generated due to aerobic metabolism influence the cellular function and their excess, if not balanced by antioxidants, may disturb the intracellular milieu [43].

Although there is some evidence for the role of reactive oxygen species (ROS) in the pathophysiology of infertility and assisted fertility [44], the existing data are conflicting and the effect of oxidative stress on the outcome of IVF is not clear [45].

Our research of a group of idiopathic infertile patients showed that concerning embryo quality and pregnancy the prediction was only observed for enzymatic antioxidants in FF. Several enzyme systems and nonenzymatic factors maintain the redox state of cells. Some authors reported that ovarian stimulation efficiency in infertile patients and pregnancy success is correlated with the elevated total antioxidant concentrations [46]. Many oxidative biomarkers have been investigated. Among them, the glutathione system with antioxidant enzymes such as GPX, GR, and GST are strongly engaged in maintaining intra- and extracellular redox balance [29, 37, 47]. The study by Nunez-Calonge has shown that the follicular fluid concentrations of these antioxidant enzymes are directly associated with higher oxidative stress levels in young patients undergoing ovarian stimulation cycles compared with fertile oocyte donors and high-response patients [41]. Decreased GPX concentration has a negative impact on fertilization rate as showed by Paszkowski et al. [48]. Our investigations of FF showed that GPX increase is associated with high-quality embryo and a higher chance of pregnancy (termed as increasing hCG levels in consecutive days). In particular, our results accordingly support this trend providing evidence that GPX levels are positively correlated with probability of the good embryo quality achievement (see Table 2 and Figures 2 and 3).

Our results indicate that higher GR, CuZnSOD, and GST decrease the embryo quality and are associated with lower pregnancy chance. The work by El Mouatassim et al. [49] showed that CuZnSOD is highly expressed in metaphase II human oocytes which indicates its role in oocyte maturation.

The report by [50] suggests that GST is involved in the normal development of oocyte and follicle maturation with lower activity found in mature oocytes (compared to immature ones) both in donors and IVF patients. Additionally, oocyte growth and maturation appear to be affected by nutritional imbalances, hormonal disturbances, and physical conditions of the microenvironment, such as oxidative stress [43]. The latter facts might affect our findings of inverse relationships of the oxidative levels measured by GR, GST, and copper-zinc superoxide dismutase in follicular fluid with pregnancy success (Table 2, Figures 6 and 7). It is necessary to highlight that early preimplantation development of the embryo is strongly dependent on oocyte quality, especially up to the 3rd day of development [51]. Thus, the pool of antioxidants stored in oocyte during oogenesis creates the system used by embryo to defend against ROS [49]. Nevertheless, some evident unexplained perturbations might have a place in embryo development with antioxidant enzymes since they were measured in FF.

### 4.3. Strengths and Limitations

In one study, we measured over 30 different biomarkers. The FF analysis and embryo assessment was done in the way of one-to-one comparison. Our study group is not numerous (53 patients); however, it is comparable for diagnosis, sperm analysis results, and the duration of infertility. General characteristic (Table 1) confirms no specific differences between patients, even if the variation of ranges of biomarkers seem to be apparent.

We are aware that the greatest verification of FF biomarkers influence on embryo quality could be achieved when all FF from every obtained follicle with MII oocyte would be assessed. This kind of study structure would allow to find existing differences between every sample of FF in the same patient. In our study, the number of retrieved oocytes ranged between 3 and 20. Additional information could be obtained if we measured biomarkers also in FF of immature (MI (metaphase I) and GV (germinal vesicle)) and degenerated ones. In this paper, we do not follow further results; live birth ratio as “take home baby” ratio depends on many additional factors, from ROS influence on the endometrium [43] to obstetrical ones.

Combining all our ROS results together, we do not have enough evidence to make any affirmation on the question of why an elevated oxidative level (measured by antioxidative GR, GST, and copper-zinc superoxide dismutase in follicular fluid) has a positive impact on pregnancy success. Considering all the negative effects of ROS on DNA, RNA, etc. and that high antioxidant levels may help improve embryo quality, the only sensible conclusion is that embryos do synthesize antioxidant enzymes themselves even in in vitro conditions that may avoid some of the physiological mechanisms for maternal selenium transfer regulation. Therefore, we tend to believe that the effects of selenium on embryo development are mainly due to the effects on genomic stability, intracellular ceramides synthesis, and peptides trafficking (as concluded in [52]), rather than because of all antioxidant activity. In fact, in the article [53], it has been shown that in vivo other complementary antioxidant mechanisms were upregulated by selenium supplementation, but not the GPX system.

## 5. Conclusions

Our results indicate that FF markers may have some advantages in predicting embryo quality and pregnancy over AMH. Statistically significant results were found only for biomarkers in FF. The GPX system seems to be mostly related to embryo quality and pregnancy. This finding should lead to further research as it seems that not only serum biomarkers and morphological assessment of cultured embryos but also other tools are necessary for achieving better embryo grading systems or at least improving the existing ones. Although the presented analysis did not show a high predictive value, oxidative stress markers in FF such as GPX could support the decision making processes in choosing the embryo for a transfer, thus shortening time to pregnancy. We plan to conduct our research in culture media and intend to do further research to determine if embryos regulate oxidative stress, how it may change, and how it may influence the implantation rate.

## Figures and Tables

**Table 1:** Hormones (in serum and follicular fluid) and enzymatic and nonenzymatic oxidative stress markers (in follicular fluid). Differences between top quality and nontop quality embryos and pregnancy success and failure for analysed risk factors were found.

Risk factor	NTQ *n* = 6	TQ *n* = 47	*P* value	Failure *n* = 37	Success *n* = 16	*P* value
Age	32.0 ± 3.16	34.8 ± 3.95	0.1725	34.7 ± 4.33	34.3 ± 3.24	0.7996
BMI	22.1 ± 4.13	22.4 ± 2.57	0.9055	22.2 ± 2.90	22.1 ± 1.48	0.8789

Serum						
AMH	4.21 ± 2.55	4.07 ± 2.75	0.9240	3.51 ± 2.12	5.30 ± 3.49	0.1805
FSH	7.83 ± 1.86	6.87 ± 1.86	0.3909	7.43 ± 1.65	5.83 ± 1.84	0.0397
LH	5.98 ± 1.95	6.44 ± 2.28	0.6848	6.27 ± 2.26	6.55 ± 2.28	0.7771

Follicular fluid						
Estradiol (pg/mL)	492375 ± 177179	487560 ± 340745	0.9655	439549 ± 285933	635900 ± 416595	0.1979
Progesteron (ng/mL)	11838 ± 2254	11837 ± 4619	0.9995	12615 ± 4838	10165 ± 3311	0.1045
AMH (ng/mL)	3.30 ± 1.70	3.50 ± 2.07	0.8396	3.42 ± 1.87	3.52 ± 2.38	0.9064
FSH (mIU/mL)	4.99 ± 1.99	4.41 ± 1.76	0.6149	4.60 ± 1.80	4.00 ± 1.62	0.3556
LH (mIU/mL)	0.73 ± 0.58	0.68 ± 0.66	0.8781	0.66 ± 0.58	0.78 ± 0.80	0.6769
Vitamin D total (ng/mL)	20.0 ± 10.4	28.2 ± 12.6	0.2167	28.1 ± 11.7	25.8 ± 16.1	0.6898
Protein g/L	57.5 ± 2.78	56.8 ± 3.18	0.6623	56.9 ± 3.35	56.2 ± 2.93	0.5737
SH *μ*mol/L	240 ± 20.8	232 ± 37.5	0.5723	235 ± 33.4	227 ± 45.1	0.6340
SH *μ*mol/g protein	4.17 ± 0.29	4.09 ± 0.60	0.6551	4.12 ± 0.54	4.02 ± 0.70	0.6927
CER mg/dL	34.2 ± 7.55	36.2 ± 6.61	0.6394	35.6 ± 6.74	35.8 ± 7.34	0.9434
TAC *μ*mol/L	0.93 ± 0.05	0.91 ± 0.07	0.5861	0.93 ± 0.07	0.88 ± 0.06	0.0736
TOS *μ*mol/L	1.68 ± 0.84	2.36 ± 0.83	0.2869	2.35 ± 0.89	2.06 ± 0.64	0.3007
GR IU/L	18.4 ± 1.54	20.3 ± 3.78	0.1037	20.5 ± 3.27	18.3 ± 1.84	0.0206
GR IU/g protein	0.32 ± 0.03	0.36 ± 0.06	0.0617	0.36 ± 0.06	0.32 ± 0.03	0.0283
SOD NU/mL	15.5 ± 0.95	16.0 ± 2.36	0.4874	15.9 ± 1.14	15.1 ± 1.44	0.1635
MnSOD NU/mL	6.19 ± 0.48	6.87 ± 0.80	0.0554	6.79 ± 0.78	6.96 ± 0.88	0.5958
CuZnSOD NU/mL	9.31 ± 1.28	9.09 ± 2.41	0.7913	9.07 ± 1.17	8.14 ± 1.12	0.0446
LPS RF	678 ± 196	624 ± 66.4	0.6231	626 ± 77.6	612 ± 45.8	0.5067
MDA *μ*mol/L	1.80 ± 0.19	1.83 ± 0.47	0.8164	1.79 ± 0.28	1.85 ± 0.73	0.8122
GPX IU/L	418 ± 36.3	472 ± 50.0	0.0487	459 ± 38.7	487 ± 58.1	0.1938
GPX IU/g protein	7.30 ± 0.81	8.33 ± 0.90	0.0766	8.11 ± 0.87	8.65 ± 0.77	0.0907
GST IU/L	3.03 ± 1.01	2.70 ± 0.76	0.5704	2.88 ± 0.85	2.28 ± 0.42	0.0113
GST IU/g protein	0.05 ± 0.015	0.05 ± 0.013	0.6020	0.05 ± 0.015	0.04 ± 0.006	0.0106
OSI	0.18 ± 0.09	0.26 ± 0.09	0.2775	0.26 ± 0.10	0.23 ± 0.07	0.4802

SH: sulfhydryl group; CER: ceruloplasmin; TAC: total antioxidant capacity; TOS: total oxidant status; LPS: lipofuscin; MDA: malondialdehyde; OSI: oxidative stress index; SOD: superoxide dismutase; MnSOD: manganese superoxide dismutase; CuZnSOD: copper zinc superoxide dismutase; CAT: catalase; GPX: glutathione peroxidase; GR: glutathione reductase; GST: glutathione-S-transferase; NU-nitric unit (1NU of SOD activity means that it inhibits nitric ion production by 50%); RF: relative fluorescence lipid extract.

**Table 2:** Hormones and antioxidants impacting embryo quality and pregnancy success.

Clinical event	Risk factor^∗^	OR (CI 95%)	*P* value
Good-quality embryo	GR (IU/L)	1.25 (0.85, 1.86)	0.263
	CuZnSOD (NU/mL)	1.14 (0.65,2.00)	0.664
	GST (IU/L)	0.61 (0.17, 2.11)	0.441
	GPX (IU/L)	1.03 (1.00, 1.06)	0.026
	GPX (IU/g protein)	3.94 (1.13, 13.7)	0.031

Pregnancy success	GR (IU/L)	0.71 (0.52, 0.97)	0.034
	CuZnSOD (NU/mL)	0.49 (0.25, 0.96)	0.036
	GST (IU/L)	0.22 (0.06, 0.81)	0.022
	GPX (IU/L)	1.01 (1.00, 1.03)	0.109
	GPX (IU/g protein)	2.29 (0.89, 5.88)	0.085

^∗^GPx: glutathione peroxidase; GR: glutathione reductase; CuZnSOD: copper zinc superoxide dismutase; GST: glutathione-S-transferase.

## Data Availability

The [patients' data, results of measurements in serum and follicular fluid] data used to support the findings of this study are available from the corresponding author upon request.
